# Sugars in peach fruit: a breeding perspective

**DOI:** 10.1038/hortres.2015.67

**Published:** 2016-01-20

**Authors:** Marco Cirilli, Daniele Bassi, Angelo Ciacciulli

**Affiliations:** 1 Department of Agricultural and Environmental Sciences (DISAA), University of Milan, via Celoria 2, 20133 Milan, Italy

## Abstract

The last decade has been characterized by a decrease in peach (*Prunus persica*) fruit consumption in many countries, foremost due to unsatisfactory quality. The sugar content is one of the most important quality traits perceived by consumers, and the development of novel peach cultivars with sugar-enhanced content is a primary objective of breeding programs to revert the market inertia. Nevertheless, the progress reachable through classical phenotypic selection is limited by the narrow genetic bases of peach breeding material and by the complex quantitative nature of the trait, which is deeply affected by environmental conditions and agronomical management. The development of molecular markers applicable in MAS or MAB has become an essential strategy to boost the selection efficiency. Despite the enormous advances in ‘omics’ sciences, providing powerful tools for plant genotyping, the identification of the genetic bases of sugar-related traits is hindered by the lack of adequate phenotyping methods that are able to address strong within-plant variability. This review provides an overview of the current knowledge of the metabolic pathways and physiological mechanisms regulating sugar accumulation in peach fruit, the main advances in phenotyping approaches and genetic background, and finally addressing new research priorities and prospective for breeders.

## Introduction

Breeding programs of peach and nectarine (*Prunus persica* L.) have traditionally focused on the selection of traits associated with fruit appearance and textural attributes.^1^ Fruit size, color, and firmness represent crucial components for retailers, making peaches attractive for consumers, resistant to postharvest handling, and allowing an extended shelf-life.^2^ Despite the significant improvements by breeding of such characters and the large number of newly released cultivars, in recent decades, a general decrease of peach consumption in the USA and European countries is observed.^3^ Poor or inconsistent flavor quality has been often recognized as the major limiting factor for acceptance by the consumers, shifting preferences toward other more flavorful fruit types. The peach cultivars present on the marketplace are often judged flavorless and appear to lack the strong ‘peach flavor’ expected by consumers, even when harvested at optimum maturity stage.^4,5^ The improvement of peach quality represents a crucial aspect for promoting consumption, prompting breeders toward the selection of novel and more flavorful cultivars^6^ that can develop flavors before the onset of the softening process. Many cultivars released in the last twenty years show important progress in flavor, attested by the introduction of the ‘low-acid’ (LA) trait, which characterizes cultivars, such as Big Top, which has a markedly sweet taste, due to both the low level of acidity and a medium to high sugar content.^7^ Although LA cultivars have been mainly developed for Asian market preferences, a general trend to increase peach sweetness has also been noted in important USA and European breeding programs.^8,9^ For example, among more than 300 cultivars released in the USA during the last decade, approximately 20% feature high soluble solids (more than 15° Brix), of which only 16% are early ripening, almost exclusively nectarines.^10^ Important international research projects are focusing on the development of new molecular tools to elucidate the genetic bases of important fruit quality traits, such as the projects RosBREED and FruitBreedomics.^11,12^

## Role of sugars in peach quality

For practical purposes, sugar content is estimated through the measure of the soluble solids concentration (SSC%, ° Brix). Although the sugar content is significantly correlated with SSC, the *R*^2^ values are often variable, ranging from 0.33 to 0.72, depending on the contribution of other optically active compounds, such as pectins, salts and organic acids.^13–15^ However, SSC is a reasonable surrogate measure of sugar content and the overall evaluation of peach fruit quality.^16^

Flavor is a combination of taste, aroma and mouth-feel attributes, defining a specific sensory profile that ultimately affects consumer judgment regarding the overall quality of fruits. In peach, taste largely depends on the water-soluble compounds, such as sugars and organic acids, conferring a sweetness and/or sourness sensation, and phenolic compounds, conferring astringency or bitterness.^17^ Sugars represent a fundamental component of fruit edible quality, predominantly conferring sweetness, one of the main attributes influencing the degree of consumer satisfaction for peaches.^18,19^ The sweetness intensity depends on the overall sugar amount as well as on the specific sugar profile (the relative content of each individual sugar) because fructose, glucose and sorbitol have approximately 1.7, 0.8 and 0.6-fold the sweetener power of sucrose.^20^ The early findings of Robertson and Meredith^21^ suggest that low-quality peaches contain lower fructose and higher sorbitol and glucose compared with high-quality peaches. However, the level of acidity strongly affects the sweetness perception. As demonstrated by the comparison of the chemical analysis and sensory profiles, sweetness is mainly correlated with the ratio between sugars and acids, the overall organic acids concentration, the amount of citrate and shikimate and fruit juiciness.^22–26^ Sugars also affect mouth-feel attributes and aroma perception; in particular, sucrose and sorbitol are highly correlated with the overall taste and aroma.^24^

A series of early studies associated SSC levels with increased consumer acceptance or lack of flavor.^22,27^ The degree of liking for peaches widely depends on the ripe soluble solid concentration (RSSC), which is the SSC of ready-to-eat peaches.^28,29^ The EU has established a minimum of 8° Brix for the peach market (Commission Regulation No. 1861/2004), despite many authors suggesting the introduction of higher SSC values, depending on the acidity levels.^30–32^ However, the establishment of a universal and reliable SSC value, assuring a minimal level of consumer satisfaction, is a difficult task. In general, consumer acceptance tends to increase along with RSSC levels, particularly when this parameter is low.^33^ In normal acidic cultivars, consumer acceptance increases rapidly with RSSC until a threshold value, specific for each cultivar, is exceeded; then, a further increase does not produce significant changes. In LA cultivars, the relationship between consumer acceptance and RSSC is more linear, progressively increasing until approximately 100% satisfaction at maximum RSSC values.^28,29^ In addition, the optimal sugar contents for peaches vary depending on the consumer group involved.^34^ Many researchers highlighted the importance of cultivar grading based on their dominant organoleptic characteristic, then developing a reliable minimum quality index within each group, thus helping to match fruit characteristics to consumers’ specific requirements.^35^

## Sugar accumulation during peach fruit development and ripening

Peach fruit accumulates different types of soluble sugars and sugar alcohols, mainly sucrose (Suc), glucose (Glc), fructose (Fru) and sorbitol (Sor). Suc is the predominant sugar in the peach mesocarp at maturity, accounting for approximately 40 to 85% of the total sugars content, followed by Glc and Fru (in variable ratios), together representing approximately 10–25%, and Sor, accounting for less than 10%.^13,22,36,37^ Mature peach fruit also contains detectable amounts of other sugars, such as maltose, isomaltose, raffinose, xylose, trehalose, 1-*O*-methyl-glucoside and fucose, and the polyols galactinol, glycerol, myo-inositol and maltitol.^38^ Starch is accumulated at the early stages of fruit development and is then rapidly metabolized, becoming almost undetectable at harvest.^39^ The total sugar content increases continuously during peach development up to full maturity, remaining constant or slightly decreasing during postharvest storage.^40^ Hexoses are the most abundant sugars in immature peach fruit until the beginning of rapid growth by cell elongation (S-III), when Suc becomes the predominant type.^41,42^ An interesting exception has been reported for the cultivar ‘Allgold’, in which Suc is the main sugar, even at the early stages of fruit growth,^37^ an aspect not further investigated in peaches. The accumulation pattern of Glc and Fru is generally characterized by reduced variations along fruit development, although their content is slightly higher at the early stages and progressively declines until maturity.^38,43,44^ The Glc:Fru ratio varies depending on genotype, usually from 0.8 to 1 for most commercial cultivars and is approximately constant across fruit development stages.^45,46^ The Suc content slowly increases in young developing fruit, remaining low until the S-III stage, when the accumulation rate increases, reaching maximum content prior to the onset of ripening, during the stage of fruit growth slowdown (S-IV).^38^ Sor levels remain low throughout fruit development, although peak accumulation has been detected during the S-II to S-III transition, followed by a sharp decline during the ripening process.^38,39^ A similar peak at pit hardening was observed for other sugars, such as xylose, raffinose, maltose and galactinol in cv. ‘Dixiland’.^38^ In particular, raffinose and galactinol are key antioxidant molecules related to stress protection, and their content is highly variable among peach varieties.^47^ During postharvest storage, Suc remains constant or slightly decreases, whereas hexoses increase or decrease,^38,40^ depending on the cultivar and the storage conditions.

## Sugar metabolism in peach fruit

A considerable amount of research has been conducted on carbohydrate biosynthesis, transport and accumulation in peaches. However, functional validation of candidate genes is particularly complex due to the recalcitrance of peach to *in vitro* regeneration.^48^ Most of the knowledge at the functional level originates from other *Rosaceae* species or model plants, which provide useful information regarding the general aspects of carbohydrate metabolism, but do not allow for framing of the possible specific behavior in peaches.

Carbohydrate partitioning in peach fruit is regulated by a complex network of metabolic activities and physiological processes, including carbohydrate biosynthesis in source tissues, long-distance translocation by phloematic flux, metabolism and accumulation in fruit sink tissues. Sor and Suc are the main translocated assimilates in the phloem sap of the Rosaceae family.^49^ In mature peach leaves, Sor accounts for approximately 70% of the transported carbohydrates (approximately 2–3:1 ratio compared to sucrose, depending on cultivar), whereas Glc and Fru are present in lower amounts.^50^ The sugar unloading pathways in peach fruit tissues have not been fully elucidated nor has the primary carbohydrate source supplying the rapid fruit growth and Suc accumulation during the S-III stage. Evidence has been provided for an apoplastic Suc transfer at the S-I and S-III stages,^42^ which is also observed in apple and pear,^51,52^ and a preferential utilization of Suc in growing fruits.^53^ However, both apoplastic and symplastic routes have been proposed for sugar uptake^54^ because in other species, the unloading route varies according to the fruit developmental stage and growth conditions.^55,56^ Membrane sugar transporters regulate carbohydrate movement between compartments and may exert strict control on their fluxes. At the molecular level, peach genome availability has allowed for the identification of major sugar-transporter gene family members. The involvement of SOTs transporters in active Sor unloading pathways has been elucidated in sour cherry,^57^ and 10 *SOT* genes have been identified in peach, three of which are expressed in mesocarp tissues.^58^ Moreover, three membrane-localized Suc/H+ symporter proteins (SUTs) involved in apoplastic loading/unloading have also been characterized: *PpSUT1*, barely detectable in fruit tissues, *PpSUT2,* mainly expressed in phloematic cells and *PpSUT4*, the most abundant transcript, expressed in both parenchyma and phloem tissues.^42^ *PpSUT2* is predominantly located on the plasma membrane, suggesting a role in Suc retrieval from the symplastic continuum into the phloem. In the S-I stage, *PpSUT4* is localized in the tonoplast and has a role in the regulation of Suc release to sustain cell metabolism, whereas at the S-III stage, the expression of *PpSUT4* decreases, allowing for the start of Suc accumulation into the vacuole. In addition to sorbitol and sucrose transporters, 29 members of the hexose transporter gene families (STPs, TMTs and HTs) are also present in the peach genome, with at least 13 isoforms expressed in the mesocarp tissues.^58–60^ Recently, a novel family of conserved Suc transporters was discovered in Arabidopsis, the SWEET uniporter proteins, localized at the plasma-membrane or tonoplast.^61,62^ In peach, 15 putative SWEET coding sequences were found, two of which are expressed in the fruit, suggesting a possible conserved role in Suc distribution.^42^ Molecular and functional evidence are still missing, as for the rest of the hexose transporters families.

The sugar distribution within the cell compartments of the fruit mesocarp varies depending on the developmental stage, shifting from a predominant cytoplasmic localization in young fruit to the vacuole at maturity.^63^ Upon translocation, Suc is cleaved to Glc and Fru by sucrose synthase (SUS) and invertase (INV) enzymes.^64^ Both enzymes act on the Suc substrate, but SUS yields Fru and UDP-glucose (UDPG), enabling reversible Suc synthesis, whereas INV converts Suc irreversibly into Glc and Fru. A total of six *PpSUS* genes are present in the genome, all expressed in mesocarp tissues. *PpSUS1,* the only studied isoform*,* is prevalently expressed in fruit compared to leaves and is upregulated during fruit ripening and postharvest storage.^65^ However, the enzyme activity of SUS remains constant or only slightly increases throughout fruit ripening.^44,65,66^ Regarding the invertase gene family, six cell-wall (CWINV), two vacuolar (VINV) and eight neutral/alkaline (NINV) genes were identified, with at least three, two and seven isoforms expressed in the fruit mesocarp, respectively.^58^ CWINVs and VINVs are acid invertases (AI), localized to either the cell-wall or vacuole, whereas NINVs are neutral invertases (NI) with cytosolic localization.^66^ In model plants that import and metabolize sucrose alone, extracellular invertase plays a pivotal role in sugar partitioning by facilitating apoplastic phloem unloading.^68–70^ For example, a lack of acid invertase activity characterizes the sucrose-accumulating species *S. chmielewski*, and the silencing of the vacuolar invertase gene *TIV1* in tomato increases sucrose, thereby reducing the hexoses content.^71,72^ In peach, the physiological relevance of invertase activity has not be fully addressed. Both AI and NI activities strongly increase during the S-I to S-II transition, remaining stable until ripening and after harvest.^38^ In contrast, the capacities of NI and AI are stable throughout fruit development in a BC2 inter-specific [SD × Summergrand] × Zephyr population (S × Z).^44^ Expression studies of four neutral invertase isoforms (*PpNI1-4*) suggest a major role for *PpNI2*, whose transcript strongly increases at the S-III stage concomitantly with the rise in Suc accumulation, whereas *PpNI1* is mainly expressed during the early stages of peach development.^38,73^ Molecular evidence regarding acid invertases is limited to the *PpCWINV1* isoform, mainly expressed in leaves.^66^ As suggested in apple,^74^ and therefore also conceivable for sorbitol-transporting species, such as peach, Suc hydrolysis by invertase may play only a minor role in fruit sugar assimilation.

In fruit, Sor can be rapidly oxidized into Fru by NAD-dependent sorbitol dehydrogenase (SDH) or sorbitol oxidase (SO).^39^ Despite SO activity being detected in peach fruit,^75^ it plays only a minor role in Sor metabolism.^76^ SDH is encoded by seven genes in peach, with at least four detected in fruit.^58^ The expression pattern of one *PpSDH* gene during peach development showed two peaks, at the end of the S-I stage and during fruit ripening, which is in agreement with the rates of SDH activity.^38,39,77^ Comparing the commercial and native peach accessions showing high and low Fru content, respectively, Kanayama *et al.*^78^ suggested that the metabolic capacity of SDH might be responsible for the different Fru levels in fruits. However, this hypothesis was not confirmed because no significant difference in SDH activity was observed in progeny segregating for Fru content.^44^

The pool of Glc and Fru resulting from sucrose and sorbitol metabolism can be stored in the vacuoles or phosphorylated to glucose-6-phosphate (G6P) and fructose-6-phosphate (F6P) by hexokinase (HK) and fructokinase (FRK), whereas the interconversion between F6P, G6P, UDPG and glucose-1-phosphate (G1P) is catalyzed by phosphoglucoisomerase (PGI), phosphoglucomutase (PGM) and UDP-glucose pyrophosphorylase (UGP) by readily reversible reactions.^79^ G1P enters the starch biosynthesis pathway, whereas F6P can enter the glycolytic pathway or can be combined with UDPG to synthesize Suc by sucrose phosphate synthase (SPS) and sucrose-phosphate phosphatase (SPP) through an essentially irreversible reaction.^80,81^ The enzymes PGM and UGP, which are linked to hexose phosphates metabolism, displayed the highest capacities,^44^ although the low G1P and starch contents in peach fruit suggest that UGPase activity may be directed toward UDPG biosynthesis. HK and FRK capacities are higher during the final stage of peach fruit development in genotypes with high Glc:Fru ratios.^44^ Among the four putative *SPS* genes identified in peach, only *PpSPS1* and** *2* have been studied in detail. *PpSPS1* did not significantly change during peach fruit development, whereas *PpSPS2* was upregulated during the S-III to S-IV transition, with a further increase detected postharvest.^38,66^ In contrast, SPS activity increased only during the early stages of fruit development.^44^

Collectively, the wide number of studies analyzing the gene expression and activities of key structural enzymes does not provide a clear framework that explains sugar accumulation patterns in peach fruit. As demonstrated by extensive analysis of sugar-related enzymes,^44^ temporal variations in enzymatic activities are too small compared to the broad changes in metabolite concentrations, revealing a remarkable stability across years and genotypes with variable sugar compositions. Genome-wide data on the transcriptional profiling of both sugar transporters and structural genes along peach fruit development are not available; thus, the role of different isoforms belonging to multigenic families is unclear.

## Phenotypic and genetic variability of the sugar content in the peach germplasm and breeding populations

In many crops, wild accessions have been proven to be a useful source for the introgression of important traits associated with fruit quality and/or for the mapping of the genomic regions undergoing strong selective pressure during domestication. This aspect could be of particular importance for *P. persica*, characterized by a narrow genetic base and low intraspecific diversity.^82,83^ The comparison of fruit composition among peach cultivars, *P. davidiana* (the wild-related species more distant to peach) and hybrids, suggests that peach domestication and hybridization have resulted in a large increase of sugar content.^84^ In *P. davidiana,* the fruit sugar content is significantly lower than peach (<100 mg/g of dry weight), Suc is predominant and Fru is higher than Glc. Information regarding the variability of the sugar composition in F1 peach × *P. davidiana* hybrids is limited to a few Summergrand × P1908 selections (SD) showing a total sugar content similar to peach and higher Glc and Fru content with respect to peach parentals.^84^ In BC2 interspecific [SD × Summergrand] × Zephyr (S × Z) progenies, the Glc:Fru ratio is approximately 1:1 in most seedlings, despite approximately 10–15% of the individuals showing transgressive segregation for high Glc content compared to peach parentals.^85,86^ In other wild species, *P. davidiana var. potaninii*, *P. mira*, *P. kansuensis* and *P. ferganensis,* the fruit sugar composition remains largely uncharacterized, as with the rest of the Eastern Asia germplasm. Recently, comparative population genomic studies have begun to explore the genetic variability associated with landraces, ornamental and wild species belonging to the Chinese peach germplasm, providing rough information regarding the fruit SSC, ranging from 7.7° to 17.5° in *P. persica* and 10° to 16° Brix in wild accessions (Supplemental File 1).^87–89^ The main commercial Chinese peach cultivars show lower inbreeding levels and a greater genetic diversity compared to Western cultivars (notably from the USA, and European and USA-derived).^90^

The sugar composition of many commercial, native and ornamental peach cultivars of Japanese germplasm was investigated by Moriguchi *et al.*^36^ Suc was the predominant sugar in native and ornamental peaches, with similar amounts compared to commercial peaches. Moreover, Japanese and Western cultivars were characterized by nearly equal percentages of Fru and Glc, contrary to native and ornamental peaches, which are characterized by a high Glc:Fru ratio and Sor amounts (Supplemental File 1). The majority of works regarding sugar composition and SSC variability have been conducted on European and USA cultivars (Supplemental File 1).^13,21,22,90^ Detailed information is available for a limited number of cultivars, making it difficult to unbundle the environmental effects and, thus, provide a more complete overview of intra- and intercultivar variability. Reig *et al.*^9^ evaluated the SSC and sugar composition of 108 recently developed peach cultivars from public and private USA and European breeding programs, reporting a wide range of variability for SSC (9.5°–19.8° Brix) and total sugar content (89.1–184.5 gL^−1^). Font i Forcada *et al.*^91^ comparing 94 traditional Spanish (mostly non-melting flesh types) and worldwide accessions, reported extensive variability of both the overall sugar content and composition. The total sugar content varied from 63 to 136 g/kg FW (Suc 35–98, Glc 4–15, Fru 2–14, and Sor 2–35), whereas SSC ranged from 12° to 18° Brix. However, in a survey of 120 Eastern and Western cultivars released during the last century (mainly from Japan, Korea and the USA), reduced variability in fruit SSC was reported, ranging from 9.1° to 13.9° Brix, with significant but very low differences between Eastern and Western cultivars (overall average values of 11.7° and 10.8°, respectively).^92^

The variability of the sugar contents and their profile has been evaluated in many intraspecific and interspecific progenies (Table 1).^1,14,37,44,85,93–95^ The analysis of traits of interest in segregating populations allows estimation of the crucial parameters for the establishment of an effective breeding strategy, such as environmental effects, phenotypic frequencies and heritability. Overall, seedlings from different cross populations exhibited wide phenotypic variability for the total sugar content (from 60 to almost 140 mg/g FW) and for individual sugars and a broad range of SSC (from below 9 to over 17° Brix), suggesting that there is genetic potential to improve the sugar content and composition in commercial peaches. All traits displayed continuous variation, following a normal or bimodal distribution among progenies, which is typical of quantitative or polygenic inheritance. Depending on the season, phenotypes can significantly deviate from these distributions. Suc was the predominant sugar in almost all progenies, whereas Glc and Fru ranged within 0.4–2.5. Sor was also variable within progenies, although the range of variability rarely exceeded the Glc or Fru amount. Although the mean values of each sugar were variable in the progenies within the parental range, a remarkable percentage of transgressive individuals with higher content with respect to either parental has been reported in almost all populations. Such transgressive segregation indicates that it could be possible to select for high sugar content in most seedling populations. Some works provided a rough estimate of sugar heritability.^37,96^ A moderate to high broad sense heritability has been calculated for SSC (0.33 to 0.72), based on 13 genotypes and approximately 2000 observations, and low heritability for both total and individual sugar (approximately 0.20, except for Sor, 0.50).^37,96^ In contrast, high heritability of Suc, Glc, Fru, Sor and total sugars, ranging between 0.65 and 0.90, was estimated in a reciprocal cross ‘Zaoxing’ (an LA flat peach) × ‘Zaolupan’ (a non-LA round peach), despite the size of the progenies being insufficient to provide a reliable evaluation.^46^ Interestingly, Wu *et al.*^46^ demonstrated that maternal inheritance did not affect the sugar composition.

## Field and environmental factors affecting the sugar content and profile

A large proportion of phenotypic variability for the peach sugar content depends on environmental factors and genotype-by-environment interactions. Fruit sugar variations in different growing seasons and locations, within trees of the same orchards, within the same tree and also within the fruit itself, are not negligible in comparison with the variation between genotypes.^97^ As described before, the sugar content varies depending on the fruit developmental stage and is mainly regulated by carbohydrate supply, metabolic transformation and dilution effect due to variation in fruit volume.^98^ These physiological and metabolic processes are influenced by field practices, such as irrigation,^99,100^ fertilization,^101,102^ rootstock:scion interactions,^103–105^ training system,^106^ pruning and canopy management.^107,108^ Additionally, environmental variables, such as temperature, solar radiation, photoperiod, precipitation and soil patterns, influence the tree-growing environment and have been widely proven to result in wide variations in sugar accumulation.^16^ The variability of the sugar contents between trees is smaller compared to the within-tree variability, which reaches differences up to 10° Brix.^109^ Sources of within-tree variability have been mainly ascribed to fruit position in the tree, microclimatic gradients inside the canopy, leaf to fruit ratio and the vigor of fruit-bearing shoots. These factors mainly affect the availability of carbohydrates supporting fruit growth.^110^ Nevertheless, even under condition of unlimited carbohydrate availability, as in thinned trees, differences in fruit sink activity result in substantial variations in fruit sugars.^111,112^ Not negligible, the SSC variability within a single fruit can be as high as 4° Brix, with differences commonly found between the blossom/stem-end and between cheeks/suture.^113,114^ For these reasons, the accuracy of the SSC and sugar content estimation could be improved by evaluating a larger number of fruits within a single tree rather than by increasing the number of trees *per* genotype.^85^

Climate and crop load are considered the most important sources of year-to-year variability.^115^ The relationship between yield and SSC is generally negative, although the degree of correlation varies depending on genotypes.^1,14^ High crop load has been negatively associated with carbohydrate accumulation in fruits because it alters the source–sink balance, causing an increase of sink competition among fruits.^116^ Several authors have reported contradictory results regarding the seasonal variability of the sugar composition.^1,37,85,86^

The effects of environmental conditions and field practices on sugar accumulation in peaches have been deeply investigated, helping to explain the major causes of within tree variability in fruit composition. Collectively, the authors agree that the SSC, total and individual sugar contents are strongly affected by seasonal variability, in contrast to the sugar profile, which is relatively constant across environments and genotypes. However, how the environment affects the metabolic fluxes at both the enzymatic and gene expression levels remains to be elucidated.

## Modeling sugar accumulation in peach fruit

Sugar accumulation in fruit is a complex quantitative trait, affected by environmental conditions and dependent on many interconnected physiological and metabolic processes, and controlled by multiple genes that interact with the environment and crop management. Quantitative trait loci (QTL) underlying the traits of quantitative nature often explain low phenotypic variability, with reduced stability across years and strong genotype-by-environment or genotype-by-management interactions. The virtual profiling of phenotypes through ecophysiological process-based simulation models (PBSM) represents a promising approach to overcome such difficulties due to their ability to mimic complex systems and to integrate multiscale levels of control.^117–119^ Ecophysiological modeling relies on the analysis of parameters involved in the development of traits, instead on their direct measurement.^120^ The identification of key genetic parameters allows for simulation of the performance of genotypes in many environments because they are independent of the environment. Such parameters can be considered as genotypic traits and are more suitable for association studies compared to the direct measure of complex traits affected by the environment. In peach, the SUGAR model has been developed to simulate the variations of the sugar composition during the S-III stage of fruit development based on carbohydrate supply, changes in fruit metabolism and assimilates dilution.^76,98^ Simplified forms of the model have also been applied to simulate the refractometric index,^121^ to analyze genotypic variation of the total sugars content in a segregating population,^122^ or to account for different Glc:Fru ratios in some genotypes.^46^ The SUGAR model was implemented in a more general ‘virtual peach fruit model’, integrating submodels for carbon assimilation, allocation and water fluxes to predict the evolution of important fruit quality traits.^123^ Among the several genotypic parameters that compose the model, 10 were able to explain 52% of variability in the fruit sweetness index.^124^ In particular, hydraulic conductance of the fruit surface (*aL*) and the coefficient of the transfer function between sugars and other compounds (*k*_sugar_) are the main parameters linked to sugar content and also highly correlated with other associated with fruit growth demand and duration.^122^ Some QTLs associated with ecophysiological parameters and influencing fruit sugar metabolism, although showing a low stability across years, were identified.^125^ For example, *r*_su_ (the Suc proportion of the total sugar amount) co-located on linkage group (LG) 1 with QTLs for fresh mass and early fruit growth. QTLs for sugar content on LG2, 4, and 7, co-located with QTLs for parameters involved in fruit water fluxes, such as *aL* and ρ (permeation coefficient of the fruit surface to water vapor). QTLs for growth duration (*dd*_max_) were co-located with those for *aL*, *k*_sugar_ and *r*_su_. This preliminary study demonstrated the potential usefulness of ecophysiological models. However, to increase their applicability, the models should also include additional genetic information via genotype-dependent parameters by easily measurable physiological traits and known QTLs or, even better, genes.^126^

## Qtl-mapping of sugar-related traits in peach

The improvement of peach quality traits has been achieved through traditional phenotypic selection within the seedling populations. However, this procedure is expensive and time-consuming, requires the screening of a large number of individuals, and above all, is mostly effective in fixing highly heritable traits. The selection for increased SSC, reportedly having moderate heritability, has allowed for a certain improvement of the sugar content in medium to late ripening cultivars, despite the variations caused by environmental and field practices. The development of peach cultivars with short fruit development periods (FDP) with high SSC is difficult due to the negatively correlated selection response between FDP and SSC,^96^ although current USA breeding programs suggest the possibility to develop early-ripening peaches with moderately high SSC.^10^ In contrast, the improvement of traits related to individual sugar contents, characterized by low heritability, is more complex to achieve by conventional selection. The identification of genetic determinants controlling these traits and the development of markers closely linked to relevant QTLs might allow for a marker-assisted selection (MAS) approach by pyramiding combinations of genes and assembling target traits more precisely.

QTLs for sugar-related traits have been mapped in a large number of species, including strawberry,^127^ apple,^128^ sweet cherry^129^ and apricot,^130^ indicating some levels of synteny among *Rosaceae* species. Despite the huge amount of available data, the genetic determinants underlining these QTLs have not been identified, except in a few cases for tomato, a model species for unveiling the molecular mechanisms regulating sugar accumulation in fruit. Among the identified loci in tomato, the most important are linked to altered sugar transport, starch biosynthesis or Suc invertase activities, such as the fine-mapped Brix 9-2-5 locus, which located an apoplastic invertase.^69,131,132^ A quantitative trait nucleotides (QTN) caused by an amino acidic substitution in the LIN5 gene is responsible for the altered activity between the cultivated and wild species allozymes.^133^

**Figure 1:**
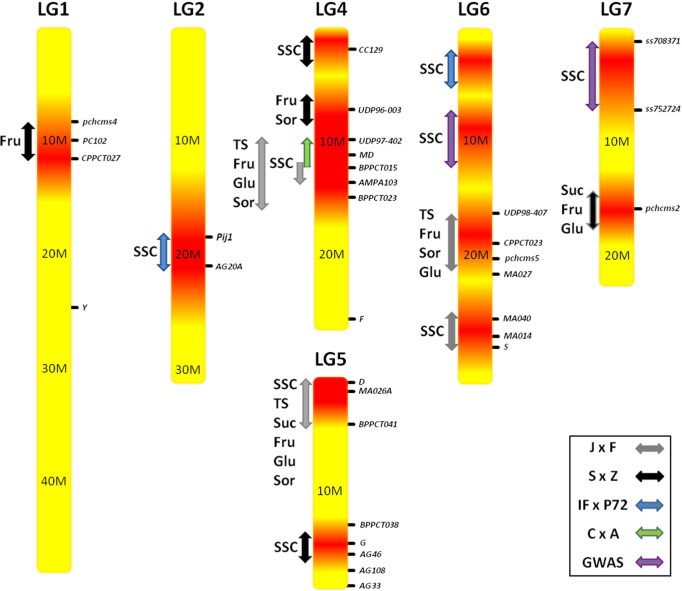
Graphical map of the position on linkage groups of the main sugar-related QTLs.

In peach, genetic determinants for some important fruit quality traits have been identified, excluding sugar-related traits, for which knowledge is still limited.^134^ QTL mapping experiments for the sugar content in peach have been conducted using biparental crosses for a limited number of genetic markers. The most significant QTLs associated with sugar-related traits currently identified in peach are shown in Table 2 and Figure 1. Early QTL mapping of an F2 progeny from a cross ‘Bailey’ × ‘Suncrest’ identified five loci associated with hexose concentrations, although the molecular markers could not be integrated in the *Prunus* reference map; therefore, information on QTLs locations cannot be used for a comparative mapping.^135^ A major QTL for the total soluble solid content was stably mapped on the central part of LG4, near the MD locus, in ‘Ferjalou Jalousia’ × ‘Fantasia’ (J × F),^136^ ‘Contender’ × ‘Ambra’(C × A),^137^ and S × Z progenies.^86^ Other QTLs were also detected on LG5 and 6, although in different map intervals.^86,136^ In sour cherry, a QTL for SSC was mapped on LG6, suggesting that this linkage group might be conserved between the two *Prunus* species.^129^ Additional loci for SSC were identified in a BC1 population derived from a cross between a peach selection ‘IF7310828’ and *P. ferganensis* ‘P72’ (IF × P72) at the top of LG6 and on the distal part of LG2,^138^ as also observed in a ‘Bolero’ × ‘Oro A’ population.^137^ Recently, an association study involving a large number of accessions belonging to the USA reference set for the peach germplasm have identified two genomic regions linked to SSC, on the central part of LG6 and at the bottom of LG7, although unstable across the two years of phenotypic observation.^139^ Many of the QTLs for SSC have been confirmed by a large association study involving over 1000 accessions within the FruitBreedomics project, albeit apart from QTLs located near the MD locus, the others have intensities close to the threshold probability (Aranzana *et al.*, 2015, unpubl. data).

Main QTL mapping experiments for the content of individual sugars have been performed on J × F and S × Z populations, identifying several genomic regions.^86,93,94,136^ However, only a few loci were confirmed across the two populations. A QTL with a main effect on the Suc content was mapped on LG5, in the region of the D locus in J × F progenies, and on the distal part of LG7 in S × Z progenies.^86,136^ QTLs linked to Glc, Fru and Sor content all seem gathered in clusters on LG4, 5 and 6. However, in the S × Z population, a major QTL for Fru content was also found on LG1 (at the FRU locus) in addition to minor QTLs affecting both Glc and Fru co-located on the distal part of LG2 and 7.^86^ Non-significant QTLs were identified for the total sugar content, except two minor QTLs located on LG5, at the D and G loci.^86,136^ Unexpectedly, Quilot *et al.*^86^ reported the presence of several favorable alleles for sugar content provided by *P. davidiana* parental, despite the low-sugar phenotype of its fruit. This aspect should be confirmed in the future, considering that the introgression of such valuable alleles in peach would require several rounds of backcrosses to eliminate the many unfavorable traits conferred by *P. Davidiana,* such as size, flavor and external appearance.

Overall, the mapping experiments suggest that both the total and individual sugar contents in peach fruit are governed by several QTLs with minor effects, often gathered in clusters. In many cases, such QTLs are unstable, due to strong environmental effects and are characterized by low LOD scores and small percentages of explained phenotypic variability. The number of loci governing sugar content is only roughly estimated, as well their genomic position, spanning regions on the order of 5–10 cM. Despite the availability of genome sequences and the attempts to identify candidate genes for the control of sugar-related traits,^140^ such intervals are too large to enable QTLs map-based cloning. The likelihood of hundreds of genes being present within these regions makes it difficult to identify the linked gene(s).

## Relationship of the sugar content with the agronomical and pomological traits

The evaluation of possible correlations between the sugar composition and physicochemical parameters is of particular importance for breeders, either to improve related traits simultaneously or to reduce undesirable side effects when selecting for one of the correlated traits. The relationships of sugar with fruit size, shape, flesh color, flesh adhesion to pit, pubescence and maturity date have been investigated.

The duration of the fruit development period (FDP) and maturity date (MD) affect the sugar contents, as broadly indicated by the reduced SSC of early-ripening fruits in comparison with mid-to-late ripening ones. A good correlation was reported between SSC and FDP (>0.60), and there was no relationship with blooming date.^96^ A significant and positive correlation between FDP and the SSC, Glc and Fru contents has been observed in a J × F progeny^94^ and in a germplasm collection showing extensive difference for maturity date.^91^ In contrast, these correlations were not evident in other progenies, although a general tendency towards higher SSC was noted in the latest ripening accessions. The correlation between ripening date and sugar content is supported by QTL co-localization on LG2^136^ and by the co-localization of a QTL for SSC at the MD locus, supporting the hypothesis of a pleiotropic effect.^137^ An NAC gene, a candidate for the control of ripening time in peaches, has been recently fine-mapped at the MD locus;^141^ however, it is unknown how it may affect sugar accumulation at the functional level.

The relationship between fruit weight (or size) and sugar content has obvious implications for fruit quality, although the relationship is complex and often not trivial. Some studies have documented a weak or not significant correlation between sugars and both fruit dry (DW) and fresh (FW) weight.^91,96,142^ For example, fruit fresh mass at harvest explained only a small amount of the total sugars variability in 14 different peach progenies.^14^ A significant relationship between the mesocarp DW, SSC and sugars was found in an S × Z population,^86^ and a negative correlation has been reported between the Glc content and both FW and DW.^85^ Although a correlation between fruit DW and sugar content is expected because carbohydrates compose approximately 60% of the DW, significant variability for sugar content has been found among fruits of similar weight because, as observed in tomato introgression lines, the proportion of DW consisting of sugars may vary depending on the metabolic utilization of the assimilates for growth or for storage.^143^ Differences in sink efficiency of individual fruits play a critical role in both DW and soluble sugar accumulation in peach.^144^ It has been hypothesized that assimilate partitioning within individual fruits depends on differences in the mesocarp cell number and size because their variability explains 77% of the variation in the Suc content between fruits within a single tree.^145^ A strong correlation between the sugar concentration and the rate of cell expansion has also been observed in peach,^146^ and in melon fruit, a preferential accumulation of Suc in larger cells has been demonstrated.^147^ Moreover, fruit cell expansion is also regulated by the water balance at the whole plant level and fruit transpiration, ultimately affecting sugar concentration via dilution.^148,149^ Crop load can significantly alter the relationship between fruit mass and SSC, particularly in high-load conditions.^150^ The correlation between fruit size and sugar content is evident when comparing peach and nectarine fruit. For example, the comparison of two peaches, ‘Tropic Beauty’ and ‘Fla.M3-1’, and their respective nectarine mutants ‘TBN’ and ‘M3-1N’, suggested that the increased sugar concentration in nectarines depends on a decrease of the fruit size compared to the original peach. Therefore, the mutation affects the fruit growth and not its ability to accumulate sugars.^151^ A higher SSC content was also observed in sibling peach and nectarine seedlings from three hybrid families,^152^ as well in the nectarine mutant ‘Yuval’ compared to its original peach ‘Oded’.^153^ The G locus, controlling the peach/nectarine trait, has been recently characterized by a variant discovery approach, identifying an LTR retro-element insertion in exon 3 of the candidate gene *PpMYB25* as the cause of the recessive glabrous phenotype.^154^ The fruit weight reduction in nectarines suggests a pleiotropic effect of this mutation on the sugar content. Alternatively, the reduced size depends on a tightly linked QTL. Nevertheless, contrasting results have been reported by mapping experiments with a QTL for fruit size and sugar content co-localized near the G locus in S × Z but not in J × F populations.^86,93^ In addition, QTLs for high Fru and Glc concentrations co-located with QTLs, showing a negative effect on fruit size on LG4, 5 and 7.^86^ The co-localization of QTLs with opposite allelic effects for sugar content and fruit weight has also been observed in tomato, suggesting that some genes may have pleiotropic effects.^155^

A general tendency for a greater sugar content in flat-peach has been observed by exploring germplasm resources.^156^ In progenies segregating for peach/nectarines and flat/round traits, SSC tends to decrease in round peach fruit compared to flat-peach and flat-nectarines, respectively.^157^ As described above for locus G, the increase of SSC may be due to the pleiotropic effects of size reduction because locus S, controlling the flat-shape trait, is associated with a QTL for fruit weight on LG6 in a J × F progeny.^93^

A significant association between the sugar composition and flesh color has not been highlighted, although sugars tend to be higher in white-flesh cultivars.^14,158,159^ The co-localization on LG1 of the FRU locus, controlling the Fru content and sweetness,^86^ with the Y locus, controlling the yellow/white color of the flesh,^160^ might explain the association of both traits.

In the case of the freestone/clingstone trait (*F* locus), slightly but significantly higher SSC, total and individual sugar contents were found in freestone fruits.^14^ However, other authors reported no significant association,^85^ or, in contrast, higher total sugars in clingstone cultivars.^91^ Although a QTL for sugar content has been reported on LG4, where the F locus is located,^86^ the difference does not depend on the linkage of the two traits but rather on the genetic background of the analyzed genotypes.

## Conclusions and perspectives

Presently, the development of new cultivars showing improved sensory attributes is a crucial task for breeders to raise the peach appeal on the fresh market. The improvement of eating quality of peach can be achieved by enhancing the sugar content or sweetness perception (for the low acid types), which are generally prized by consumers. However, as demonstrated by several studies, the increase of the sugars content is not always accompanied by a parallel increase in the liking degree. Therefore, the selection of new cultivars should be defined as a more specific target to be achieved, based on detailed information regarding consumer preferences and perceptions.

An improvement of the sugar content has been observed in recently released cultivars from different conventional breeding programs. Nevertheless, further and substantial improvements by classical breeding methods are limited by the scarce knowledge of the gene network expressing the phenotype. Although the strong influence of environmental factors and genotypic × environment interactions could mask the effects of minor QTLs, the genetic determinants regulating sugar accumulation could be identified, allowing for the identification of molecular markers to be used in marker-assisted selection (MAS) or breeding (MAB). Even when several minor QTLs are made available, marker-assisted approaches could represent the best strategy for an efficient screening procedure, through QTLs pyramiding. Unfortunately, none of the putative identified QTLs governing sugar-related traits in peach have been fine-mapped. The lack of detailed information on their effects and genome location, as well as their validation in different genetic backgrounds, prevents the development of suitable molecular markers for breeding purposes.

The availability and the high level of synteny among several *Prunus* genomes, together with a wide array of new technologies, offers unheard-of opportunities to breeders. NGS technologies have been implemented in linkage mapping experiments to develop novel and promising approaches, such as RAD-Seq,^161^ GBS,^162^ GS,^163^ and QTL-Seq.^164^ These powerful approaches could allow for the creation of high-resolution maps with a high number of molecular markers. However, these tools could suffer the same limitations as classic linkage mapping strategies because the increment of markers density is often not sufficient for unambiguous QTLs identification. Map resolution can be limited by the few recombinant events occurring in early generations and more often by the small size of the segregating progenies. The increase of progeny size is often difficult to achieve due to the time and the costs of field management. Alternative strategies of QTL mapping based on advanced backcross, NILs, RILs or double haploids, are not easy to perform in a tree crop. Furthermore, genome-wide association studies (GWAS) are ineffective in peaches, at least when applied to the European and USA germplasm, which are characterized by narrow genetic bases and high levels of linkage disequilibrium (LD).^165^

To exploit the full potential of the above described technologies and to increase the genetic gains through so-called precision breeding,^166^ the availability of accurate and high-throughput phenotyping tools is of paramount importance. Although data collection of sugar contents is not difficult from a technical standpoint, the phenotyping is hindered by the strong environmental effects and the high within-tree variability, in turn representing another reason for the lack of accuracy of the QTLs mapping experiments. A full understanding of the processes underlying the interconnections among yield, fruit growth, dry and fresh mass accumulation and sugars partitioning is essential in the environmental variability and its interaction with genotype. Ecophysiological modeling represents a viable approach to improve the phenotyping of complex traits; however, such models are currently in progress and are rarely applied by breeders because they depend on parameters that are not easily measurable.

In conclusion, the following priorities should be followed for the improvement of sugar content in peach fruit by a genetic approach: deepening the knowledge of the physiological and metabolic processes regulating sugar accumulation with the aid of new ‘omics’ tools; developing innovative phenotyping methods to address the strong environmental and genotype × environment effects, even enhancing the accuracy and simplifying the application of ecophysiological models; exploration of the phenotypic variability associated with sugar-related traits, for example, within the Far-East germplasm, to increase the genetic variability and the resolution power of association studies; improvement of the efficiency of *in vitro* transformation for the functional validation of candidate genes; defining more specific targets to be achieved for sugar levels based on fruit type, consumer preferences and perceptions.

## Acknowledgments

This work was supported by MASPES (Italian project aimed at apricot and peach breeding) and FRUITBREEDOMICS (grant 265582 – EC-GAUE, 7th Framework Program: the views expressed in this work are the sole responsibility of the authors and do not necessarily reflect the views of the European Commission).

## Author contributions

MC: searched and critically reviewed the available literature, wrote the paper.

DB: conceived the paper, contributed to the writing of the manuscript and critically revised it.

AC: searched the available literature, drafted and revised the manuscript.

All authors: read and approved the final manuscript.

## Figures and Tables

**Table 1:** Mean values and range of variability for the soluble solid content (SSC), total and individual sugars, detected in fruits of the most relevant analyzed cross populations available in literature

						Total Sugars		Sucrose		Glucose		Fructose		Sorbitol		
				SSC (°Brix)		mg/g FW										
Population			n	Mean	Min–Max	Mean	Min–Max	Mean	Min–Max	Mean	Min–Max	Mean	Min–Max	Mean	Min–Max	Ref
F1 ‘Venus’	x	‘Big Top’	75	13.9 ± 0.2	11.2–17.5	89.7 ± 1.6	67.4–138.9	58.4 ± 1.2	40.7–102.3	12.2 ± 0.3	8.3–23.4	12.4 ± 0.2	8.9–19.1	6.6 ± 0.5	1.7–19.5	95
F1 ‘Andross’	x	‘Calante’	19	12.2	7.6–17.5	85.9	36.0–109.4	64	28.2–84.4	6.6	2.3–14.6	10.3	3.8–16.1	5.1	0.9–10.6	14
F1 ‘Andross’	x	‘Crown Princess’	9	11.0		67		51.6		6.4		7.6		1.4		
F1 ‘Andross’	x	‘Rich Lady’	9	11.1		73.3		54.9		5.8		9.0		3.6		
F1 ‘Andross’	x	‘VAC-9511’	6	11.9		67		49.6		6.7		9.1		1.6		
F1 ‘Babygold-9’	x	‘Crown Princess’	19	9.7		69.4		53.5		5.7		7.9		2.4		
F1 ‘Babygold-9’	x	‘VAC-9510’	15	11.0		67.7		52.5		5.7		7.4		2.2		
F1 ‘O’Henry’	x	‘VAC-9514’	14	13.2		77.7		54.3		8.0		10.1		5.3		
F1 ‘O’Henry’	x	‘VAC-9515’	8	13.5		80.3		60.9		6.8		9.3		3.2		
F1 ‘O’Henry’	x	‘VAC-9516’	11	12.5		75.5		58.2		6.5		6.9		3.9		
F1 ‘Orion’	x	‘VAC-9513’	3	11.6		67.2		47.1		7.3		8.9		3.9		
F1 ‘Red Top’	x	‘VAC-9513’	37	9.9		64.0		48.4		5.6		7.8		2.1		
F1 ‘Rich Lady’	x	‘VAC-9511’	8	12.3		67.6		50.0		6.7		8.4		2.5		
F1 ‘VAC-9512’	x	‘VAC-9511’	10	11.6		77.3		57.1		7.0		9.0		4.2		
F1 ‘VAC-9520’	x	‘VAC-9517’	37	12.2		75.6		56.5		7.3		8.9		2.8		
F1 ‘Ferjalou Jalousia’	x	‘Fantasia’	nr	12.2 ± 0.4	nr	nr	nr	72.9 ± 2.5	nr	4.9 ± 0.4	nr	7.0 ± 0.3	nr	7.0 ± 0.3	nr	93
F2 ‘Ferjalou Jalousia’	x	‘Fantasia’	63	11.45	8–14	nr	nr	67.85	40–90	10.2	5–15	10.8	6–17	1.2	0.5–6	94
BC2 ‘Summergrand’	x	‘Zephir’	140	nr	10.1–19.3	92 ± 18	56–167	nr	20–150	nr	5–35	nr	2.5–25	nr	2.5–20	86, 120
F1 ‘Zaolupan’	x	‘Zaoxing’	46	nr	nr	96 ± 15	69–120	66 ± 17	34–110	13 ± 4.3	6.0–21	13 ± 3.6	6.8–19	4.3 ± 2.9	0.7–13	155
			43			86 ± 19	50–124	48 ± 16	19–81	16 ± 6.1	7.0–30	16 ± 6.9	6.7–34	6.3 ± 3.0	1.0–12	
F1 ‘Zaoxing’	x	‘Zaolupan’	89			91 ± 16	58–136	65 ± 18	30–120	12 ± 3.5	5.8–21	11 ± 2.8	5.2–19	2.8 ± 1.7	0.5–10	
			90			84 ± 18	48–124	47 ± 17	22–81	16 ± 6.1	7.1–28	16 ± 5.7	9.2–30	5.4 ± 3.4	0.6–18	

**Table 2:** Table 2 Localization and approximate genome position of the principal QTLs for the soluble solid content (SSC), total and individual sugars identified in peach. The statistical significance of the linkage between a QTL and marker is indicated according to the detection methods: *LOD score; **P-value; ***2 ln (Bayesian Factor)

Trait	LG	Statistical significance	Nearest marker	Locus	Approximate genome position	Population	Ref
SSC	2	3.3*	Pij1–AG20		Pp02:19 108 809..21 093 830	BC_1_ IF × P72	136
	4	0.0023**	CC129 (pchgms2)		Pp04:2 086 577	BC_2_ S × Z	86
	4	4.9*	BPPCT015	MD	Pp04:12 558 026..13 520 063	F_2_ J × F	134
	4	47.8–49.4*	MD	MD	Pp04:11 138 518..11 140 641	F_2_ C × A	135
	5	2.9*	MA026	D	Pp05:145 584..3 741 013	F_2_ J × F	134
	5	0.00027**	AG46-AG108	G	Pp05:14 652 809..18 294 595	BC_2_ S × Z	86
	6	2.9*	UDP98-416		Pp06:4 375 577	BC_1_ IF × P72	136
	6	4.47***	ss_629062–ss_630302		Pp06:7 918 349..12 571 791	GWAS	137
	6	3.3*	UDP98-407		Pp06:20 450 677..21 030 866	F_2_ J × F	134
	6	2.3*	MA014	S	Pp06:27 186 773	F_2_ J × F	134
	7	7.80***	ss_708371–ss_752524		Pp07:1 125 816..8 336 521	GWAS	137
Fructose	1	2.9e^−15^**	PC102	FRU	Pp01:9 959 357..12 857 908	BC_2_ S × Z	86
	4	0.0072**	UDP96-003		Pp04:8 768 343	BC_2_ S × Z	86
	4	10.5*	BPPCT015	MD	Pp04:10 497 063..14 742 215	F_2_ J × F	134
	5	3.0*	MA026A	D	Pp05:145 584..3 741 013	F_2_ J × F	134
	6	7.1*	CPPCT023–pchcms5		Pp06:20 450 677..21 030 866	F_2_ J × F; T × E	134,138
	7	0.0008**	pchcms2		Pp07:18 688 565	BC_2_ S × Z	86
Glucose	4	9.4*	BPPCT015	MD	Pp04:10 497 063..14 742 315	F_2_ J × F	134
	5	3.4*	MA026A	D	Pp05:145 584..3 741 013	F_2_ J × F	134
	6	7.9*	CPPCT023–pchcms5		Pp06:20 450 677..21 030 866	F_2_ J × F; T × E	134,138
	7	0.0045**	pchcms2		Pp07:18 688 565	BC_2_ S × Z	86
Sorbitol	4	0.0016**	UDP96-003		Pp04:8 768 343	BC_2_ S × Z	86
	4	6.6*	BPPCT015	MD	Pp04:10 497 063..14 742 315	F_2_ J × F	134
	5	2.2*	MA026A	D	Pp05:145 584..3 741 013	F_2_ J × F	134
	6	21.9*	CPPCT023–pchcms5		Pp06:20 450 677..21 030 866	F_2_ J × F; T × E	134,138
Sucrose	5	15.3*	MA026A	D	Pp05:145 584..3 741 013	F_2_ J × F	134
	7	2.4*				F_2_ J × F	134
	7	0.0088**	pchcms2		Pp07:18 688 565	BC_2_ S × Z	86
Total sugars	4	3.2*	BPPCT015	MD	Pp04:10 497 063..14 742 315	F_2_ J × F	134
	5	7.3*	MA026A	D	Pp05:145 584..3 741 013	F_2_ J × F	134
	6	3.3*	UDP98-407		Pp06:17 642 515	F_2_ J × F	134
